# COVID-19 Vaccination in China: Adverse Effects and Its Impact on Health Care Working Decisions on Booster Dose

**DOI:** 10.3390/vaccines10081229

**Published:** 2022-07-31

**Authors:** Chengwen Luo, Hai-Xiao Chen, Tao-Hsin Tung

**Affiliations:** 1 Evidence-Based Medicine Center, Taizhou Hospital of Zhejiang Province, Wenzhou Medical University, Linhai 317000, China; luocw0806@enzemed.com; 2 Department of Orthopedics, Taizhou Hospital of Zhejiang Province, Wenzhou Medical University, Linhai 317000, China; chenhx@enzemed.com

**Keywords:** healthcare workers, willingness, vaccination adverse reaction, decision regret, COVID-19 booster vaccine

## Abstract

Although many research studies have concentrated on people’s willingness to take the COVID-19 vaccine, little attention has been paid to the underlying mechanism of consent. An understanding of potential factors and mechanisms that affect the willingness to receive a vaccination can contribute information critical for containing the pandemic. This study explored the effects of post-vaccination adverse reactions on the willingness to take the booster dose and the role of decision regret. A self-administered online survey was carried out in Taizhou, China. Questionnaires were completed by 1085 healthcare workers (HCWs), 1054 (97.1%) of whom had completed two doses of the COVID-19 vaccine. Mediation analysis methodology was applied in this study. Our study showed that post-vaccination adverse reactions in HCWs could decrease their willingness to take the booster dose. Of note, HCWs who experienced adverse reactions after vaccination would be more likely to regret their previous vaccination decisions, which, in turn, further reduced their willingness to receive a booster shot. Decision regret mediated the relationship between adverse post-vaccination reactions and a willingness to take the booster dose. The findings implied inextricable relationships among post-vaccination adverse reactions, decision regret, and willingness to take the booster dose. It is suggested that notice of these post-vaccination adverse reactions should be further incorporated into vaccine communication campaigns and policy interventions advocating booster doses to improve vaccine uptake intent and increase the willingness to receive booster doses of a COVID-19 vaccine.

## 1. Introduction

In December 2019, the outbreak of coronavirus disease 2019 (COVID-19) was initially detected in Wuhan, China [1]. The World Health Organization (WHO) declared COVID-19 a worldwide pandemic in March 2020. The ongoing COVID-19 pandemic has caused a serious public health burden worldwide, placing millions of people at risk [2,3]. Based on epidemiological data, droplets from face-to-face contact during conversation, coughing, or sneezing appear to be the most common mechanism of spread [4]. As healthcare workers (HCWs) are in direct contact with COVID-19 patients, they are vulnerable to this highly infectious virus. Thus, as a response to the pandemic, safe and effective preventive vaccines were a potentially useful tool that could be used to reduce transmission rates and subsequent infections [5,6].

Recently, there have been reports of SARS-CoV-2 infection, hospitalization, or even death due to COVID-19 in some people who had completed both doses of the vaccine [7]. This poses new challenges for the regular prevention and control of outbreaks. The COVID-19 pandemic is still dangerous and, therefore, needs to be taken seriously. In addition, SARS-CoV-2 was prone to mutation, and vaccine effectiveness has decreased over time, which has been thought to have contributed to the re-emergence of the pandemic [8]. Hence, timely vaccination with the third booster dose to further increase the neutralizing antibody titer in the body can supplement and improve the declining protective efficacy of the vaccine and also protect against a new SARS-CoV-2 variant that may emerge at any time.

HCWs have a higher risk of SARS-CoV-2 infection [9,10]. Hence, the vaccination of healthcare workers can interrupt the spread of SARS-CoV-2 and induce beneficial ripple effects in the broader community, which is necessary to create herd immunity in all the groups that may contribute to SARS-CoV-2 transmission. To date, most research on booster vaccination with the COVID-19 vaccine has concentrated on the investigation of individuals’ willingness to take booster injections, with little attention being paid to the underlying mechanism. Understanding the mechanism of the willingness to receive the booster dose among HCWs is important to increase booster vaccination in the general population, which in turn can better control the pandemic.

Previous studies showed that the most commonly reported negative reactions after vaccination included pain at the place of vaccination, pain in the muscles and bones, general poor feelings, and fever [11]. Regardless of the vaccine, these adverse reactions influenced about 1/4 of the population [12]. It is a matter of great interest to investigate whether these post-vaccination adverse reactions affect people’s willingness to receive booster shots. As we know, individuals are usually faced with difficult decisions about their health and may later regret the choices they have made. Research found that an adverse physical health outcome was one of the risk factors most frequently reported to be related to decision regret [13]. Therefore, in this research, we aimed to study the relationship between post-vaccination adverse reactions, decision regret, and willingness to take the booster dose of the COVID-19 vaccine.

## 2. Materials and Methods

### 2.1. Study Design

We organized a cross-sectional online survey in the WeChat-incorporated Wen-Juan Xing platform. The target population was HCWs in a medical center in Taizhou, China. The samples included health professionals, such as doctors, nurses, and medical technicians, and administrative support workers, such as janitors, nursing aides, and dietary aides. Participants received the survey via WeChat or e-mail, and they answered the questionnaire by visiting the Uniform Resource Location (URL) or by scanning the Quick Response (QR) code on their mobile phones between 31 August and 8 September 2021. A total of 1103 questionnaires were collected. We then conducted an initial check of the data. Participants who were under 18 years old were excluded, and samples that were submitted repeatedly were discarded. We obtained 1085 valid questionnaires.

This survey study was approved by the Ethics Committee of Taizhou Hospital, Zhejiang Province, China (Approval number: K20210823). All procedures were conducted in accordance with the guidelines of our institutional Ethics Committee and in compliance with the principles of the Declaration of Helsinki. The participation of interviewees in the survey was considered to be informed consent. We did not ask for a separate written form of informed consent in order to preserve respondents’ anonymity. Information about all respondents was kept anonymous.

### 2.2. Questionnaires

The main contents of the questionnaire were as follows: (1) basic demographic information, including age, sex, education, occupation, professional title, and underlying diseases; (2) vaccination history, such as COVID-19 vaccination status and post-vaccination adverse reactions (HCWs who had completed two doses of COVID-19 vaccination were identified by a question asking whether respondents had been vaccinated against COVID-19. The three response categories were as follows: (a) none, (b) once, and (c) twice. We also asked the participants if they had any adverse reactions after vaccination, which was answered with yes or no); (3) decision regret, which includes 5 items, as follows [14]: (a) The decisions were right; (b) I regret the choices that were made; (c) I would go for the same choice if I had to do it over again; (d) the choices did me a lot of harm; (e) the decisions were wise ones (each item was answered on a five-point bipolar intensity scale. Participants evaluated the statements by circling a number from 1 (strongly agree) to 5 (strongly disagree). Items (b) and (d) were phrased in the negative to avoid acquiescence bias. After reversing the scores of these two items, the overall sum score was produced by taking the sum of the five items); (4) the willingness to take the booster dose was measured by asking whether the participants were willing to receive the booster dose of the COVID-19 vaccine.

### 2.3. Mediation Analysis

Mediation models have been widely utilized to explore the potential mechanism of an independent variable on a response variable, and whether there was a variable that mediated the above relationship [15,16]. This analysis could have important policy consequences since mediation analysis plays an important role in understanding the mechanism whereby the change in one variable potentially causes a change in another. In this study, exposure (X) comprised post-vaccination adverse reactions (yes or no); the potential mediator (M) was decision regret; the outcome (Y) was the willingness to take the booster dose of COVID-19 vaccine (yes or no).

We concentrated on the case of a binary outcome (Y) and a continuous mediator (M), and adopted the following three regression models for mediation analysis:

(1) $$\mathrm{logit}\left(P\left(Y=1\right)\right)=c_{1}+\gamma X+\delta^{T}Z+\varepsilon_{1},$$

(2) $$M=c_{2}+\alpha X+\theta^{T}Z+\varepsilon_{2},$$

(3) $$\mathrm{logit}\left(P\left(Y=1\right)\right)=c_{3}+\gamma^{*}X+\beta M+\vartheta^{T}Z+\varepsilon_{3},$$

where Equation (1) described the relation of an independent variable and a response variable (X and Y); Equation (2) characterized the relation of an independent variable and a mediator (X and M); Equation (3) summarized the relationship between the independent variable, the mediator, and the response variable (X, M, and Y); Z was the covariates, such as age and sex; γ was the total effect of the exposure on the outcome; α was the effect of the exposure on the mediator; $\gamma^{*}$ was the direct effect of the exposure on the outcome; β was the effect of the mediator on the outcome; $c_{1}$, $c_{2}$, and $c_{3}$ were the intercept terms; $\varepsilon_{1}$, $\varepsilon_{2}$, and $\varepsilon_{3}$ were the residual terms.

Mediation effects were commonly tested via a regression-based modeling approach [17,18,19]. The first step was to identify whether there was a significant relationship between X and Y. The second step was to determine whether the relation of X and M was significant. The final step was to regress Y on both X and M. Then, to test the mediation effect, we applied the joint test method [20]. This method considered the path-specific *p*-values and provided an estimate as follows.

(4) $$P=\max\left(P_{\alpha },P_{\beta }\right).$$

Thus, we could conclude that the variable M was the intermediator between the exposure and the outcome if p < 0.05.

### 2.4. Statistical Analysis

**Figure 1:**
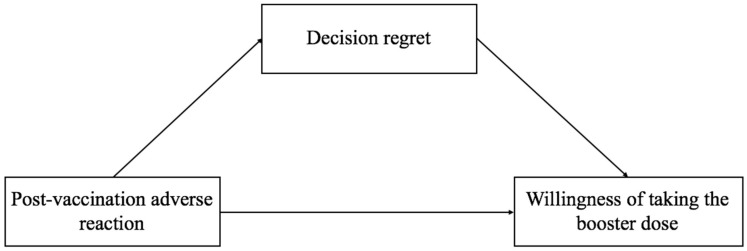
The directed acyclic graph describes the relation among post-vaccination adverse reactions, decision regret, and willingness of taking the booster dose of COVID-19 vaccine.

In this study, our main purpose was to explore the relationship between post-vaccination adverse reactions, decision regret, and willingness to take the booster dose of the COVID-19 vaccine. The framework of the above relationship was characterized in Figure 1. The exposure we considered here consisted of adverse reactions after vaccination; the potential mediator was the score of decision regret; the outcome was the willingness to take a booster dose of COVID-19 vaccine. We conducted mediation analysis based on the above mediation models and adjusted for covariates including age, sex, education, occupation, and underlying diseases.

Categorical variables of the basic demographic characteristics were presented as counts and percentages. We applied a chi-square test to initially identify the possible factors of the outcome. Finally, we adopted the three regression models (i.e., Equations (1)–(3)) to perform the mediation analysis. Variables considered statistically significant should have a *p*-value < 0.05. All statistical analyses were implemented via R software, version 4.1.0 (R Project for Statistical Computing).

## 3. Results 

### 3.1. Characteristics of the Study Participants

We obtained 1085 valid questionnaires, and 1054 (97.1%) respondents had completed their course of two COVID-19 vaccinations. Table 1 summarizes the basic characteristics of the respondents, including post-vaccination adverse reactions, age, sex, education, occupation, professional titles, and underlying disease. Among the study participants, 123 (11.7%) had adverse reactions after both vaccinations. Their average age was 34.2 ± 8.5 years old, and most were aged below 40 years old, accounting for 78.6% of study respondents. There were 165 (15.7%) males and 889 (84.3%) females. Sixty-three point six percent (63.6%) of the respondents were nurses, and more than half of the participants (67.3%) held only undergraduate degrees. The vast majority of participants did not have any underlying diseases (88.5%).

The results with respect to the incidence of willingness to take the booster dose of COVID-19 vaccine, overall and by subgroups of HCWs, are presented in Table 2. A total of 917 (87%) respondents were willing to receive the booster injection. There was a significant difference in willingness to be vaccinated between respondents with post-vaccination adverse reactions and those without ($\chi^{2}$ = 22.192, *p*-value < 0.001). For participants without adverse reactions after vaccination, 88.8% were willing to take the booster dose, while for those with adverse reactions, only 73.2% were willing to receive it. Moreover, the results of a univariate analysis illustrated that age, sex, education, occupation, professional title, and underlying disease had no significant difference in vaccination intention. However, we could observe a higher willingness to receive vaccination in some subgroups. For example, 92.9% of respondents above 50 years old were willing to receive the booster dose. Participants with junior college education levels, medical technicians, and those who had no underlying diseases also had a higher willingness to receive the booster dose.

### 3.2. Correlations between the Main Study Variables

The correlation coefficients are provided in Table 3. Post-vaccination adverse reaction had a positive correlation with decision regret (r = 0.14, *p*-value < 0.001) and a negative correlation with willingness to take the booster dose (r = −0.15, *p*-value < 0.001). Decision regret was negatively correlated with willingness to receive the booster dose (r = −0.22, *p*-value < 0.001). To conclude, the results of correlation analysis showed that the pairwise combinations of the above three variables were significant and illustrated that there was a correlation between post-vaccination adverse reaction, decision regret, and willingness to take the booster dose.

### 3.3. Testing for the Mediation Model

**Figure 2:**
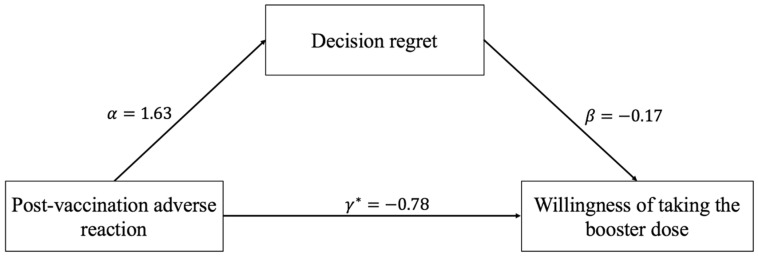
Pathway between post-vaccination adverse reaction, decision regret, and willingness of taking the booster dose. The description of α, β, and $\gamma^{*}$ can be found in Equations (1)–(3).

The results of the mediation analyses for the relationship between post-vaccination adverse reaction, decision regret, and willingness to take the booster dose, adjusting for age, sex, education, occupation, and underlying disease, are presented in Table 4. Furthermore, we also present the path-specific effects in Figure 2.

Firstly, the effect of post-vaccination adverse reaction on participants’ willingness to take the booster dose was significant (*p*-value < 0.001). Compared with participants who had no adverse reactions after vaccination, those with post-vaccination adverse reactions were less likely to receive the booster dose (OR = 0.37, 95%CI: 0.23~0.59). Hence, post-vaccination adverse reaction was a significant factor affecting the willingness to take the booster injection. Secondly, compared to respondents without post-vaccination adverse reactions, those who experienced adverse reactions had higher decision regret scores (B = 1.63, 95%CI: 0.98~2.28). The effect of post-vaccination adverse reaction on decision regret was also significant (*p*-value < 0.001).

Thirdly, the impact of decision regret on willingness to take the booster dose was also significant after controlling for post-vaccination adverse reactions (OR = 0.84, 95%CI: 0.80~0.89, *p*-value < 0.001), which suggested that participants who regretted their previous decisions were less likely to obtain a booster shot. Finally, the effect of post-vaccination adverse reaction on willingness to take the booster dose remained significant (OR = 0.46, 95% CI: 0.29~0.74, *p*-value < 0.01). All models were adjusted for potential covariates including age, gender, education level, occupation, and underlying disease. Compared to doctors, medical technicians were more willing to take the booster dose. Moreover, participants with underlying diseases had less willingness to take the booster dose than did those without. The joint test indicated that the mediation effect of decision regret on the relationship between post-vaccination adverse reactions and willingness of taking the booster dose was significant ($P=\max\left(P_{\beta_{1}},P_{\theta_{2}}\right)<0.05$). This implies that decision regret could significantly mediate the effect of post-vaccination adverse reactions on willingness to take the booster dose.

## 4. Discussion

### 4.1. Clinical Implications

The COVID-19 pandemic has dramatically impacted the lives of people worldwide and caused significant disease and economic burdens around the world. Vaccination was considered an effective and safe way to control and prevent infectious diseases. Considering that the outbreak might continue to have an influence on human beings, we may have to be prepared for ongoing vaccinations. In China, the healthcare vaccination program is well organized and high vaccination rates are expected among HCWs. However, few researchers have explored the potential mechanisms within the willingness to take the booster dose. 

This research aimed to explore the relationship between post-vaccination adverse reactions and willingness to take the booster dose and those potential mechanisms. We focused on healthcare workers who had completed their two-step vaccination. In this study, we found that individuals’ having post-vaccination adverse reactions had a negative correlation with willingness to take the booster dose. Moreover, participants who experienced adverse reactions after vaccination were more likely to regret their previous vaccination decisions. In addition, respondents who had higher decision regret scores had less willingness to receive the booster dose. The results showed that regret over previous decisions could significantly mediate the impact of post-vaccination adverse reactions on willingness to take the booster dose. To the best of our knowledge, this paper is one of the few studies on the influence of post-vaccination adverse reactions on the willingness to receive a booster dose.

Several studies have been conducted to investigate the immunogenicity, safety, and efficacy of the booster dose against COVID-19, which provides strong scientific evidence that the booster dose of COVID-19 vaccine can improve the titer and protective range of neutralizing antibodies [21,22,23]. The results of this study showed that 87% of respondents would take the booster dose, which was lower than the acceptance rate of primary vaccination reported in previous studies in China (91.3%) [24]. In addition, there have been a number of studies concentrated on the effects of different aspects of the vaccination rate [25,26,27,28]. For example, a survey conducted among Chileans found that respondents who trust COVID-19 vaccines include scientists, and medical professionals had a significantly higher willingness to accept the booster dose [25]. Moreover, compared with administrative and allied health colleagues, medical and nursing HCWs in Singapore had shorter median time to relative to receiving a COVID-19 booster [26]. Previous studies have found that, regardless of the vaccine, about 1/4 of the vaccinated population had been affected by post-vaccination side reactions [12]. Although all side reactions disappear within a week, individuals might regret the choice that they had made to receive the vaccine. Adverse physical health outcome was one of risk factors most frequently reported to be related to decision regret [13]. Both the post-vaccination side reactions and decision regret reduced the willingness to take the booster dose. Hence, in subsequent vaccine communication campaigns and policy interventions advocating booster doses of COVID-19 vaccine, in addition to the information that the vaccine is effective and safe, incorporating information about adverse reactions after vaccination is also necessary and might increase the willingness to be vaccinated.

Increasing the willingness of health care workers to be vaccinated against COVID-19 could increase the acceptance of vaccination in the general public, which closely monitors the behavior of HCWs on this issue. The attitudes of HCWs are particularly important for vaccination acceptance in the general public, since people are more likely to get vaccinated if HCWs recommend it. Another benefit of optimizing vaccination programs is the promotion of vaccination within universities and hospitals; medical authorities should focus on further providing reliable knowledge about the COVID-19 pandemic, especially with respect to adverse reactions after vaccination, and persuading and even requiring HCWs to be vaccinated against COVID-19.

### 4.2. Methodological Consideration

Some limitations of this research need to be further studied. Firstly, the sample may not be fully representative of the HCWs of China, since we only considered only one teaching hospital. In addition, survey participants were likely to be healthier than the general public, given that they were healthy enough to be employed in a healthcare institution, which might have resulted in selection bias. In addition, the vast majority of participants in this study were young, without comorbidities, and female. Further studies balancing these demographic characteristics would lead to a more accurate study. Secondly, we focused on the HCWs who have completed their twofold vaccination. However, there might be differences between HCWs and the general population. Hence, to further identify the role of decision regret in the relationship between adverse reactions after vaccination and willingness to take the booster dose, the generalization and external validity of the data and findings should be further studied. Thirdly, the online data collection method was a limitation of this study, one which could potentially lead to over-reporting or under-reporting the willingness to take the booster dose. Fourthly, our estimates were conducted at only onetime point and could not reflect long-term exposure to various factors. Further longitudinal and larger sample sizes investigations are essential not only to extrapolate findings to other regions of China but also to better understand the causal relationships.

## 5. Conclusions

In summary, our study showed that post-vaccination adverse reactions in healthcare workers could decrease their willingness to take the booster dose of a COVID-19 vaccine. Of note, HCWs who experienced adverse reactions after vaccination were more likely to regret their previous vaccination decisions, and this, in turn, further reduced their willingness to receive a booster shot. Although participant bias should be considered, these results are important data for improving the vaccination rate of the booster dose in the future. Generally speaking, post-vaccination adverse reactions disappear within a week; therefore, these adverse events should not be a significant concern for vaccination programs. Therefore, post-vaccination adverse reactions should be further incorporated into vaccine communication campaigns and policy interventions advocating booster doses, both to improve vaccine uptake intent and potentially increase willingness to receive booster doses of a COVID-19 vaccine.

## Figures and Tables

**Table 1:** Baseline characteristics of the healthcare workers (N *n* = 1054).

Characteristics	Category	Sample	
		Number	Percentage (%)
Post-vaccination adverse reaction	No	931	88.3
	Yes	123	11.7
Age (years)	<30	363	34.4
	30~39	466	44.2
	40~49	183	17.4
	≥50	42	4.0
Sex	Male	165	15.7
	Female	889	84.3
Education	Senior Secondary and below	71	6.7
	Junior college	162	15.4
	Undergraduate	709	67.3
	Graduate	112	10.6
Occupation	Doctor	174	16.5
	Nurse	670	63.6
	Medical Technician	127	12.0
	Others	83	7.9
Professional titles	Internship	18	1.7
	Primary grade	447	42.4
	Medium grade	359	34.1
	Associate professor	86	8.1
	Professor	47	4.5
	Others	97	9.2
Underlying disease	Yes	121	11.5
	No	933	88.5

Note: underlying disease includes hypertension, diabetes, cardiovascular disease, chronic respiratory disease, chronic kidney disease, chronic liver disease, and cancer.

**Table 2:** Univariate analysis of factors associated with willingness to take the booster dose of COVID-19 vaccine.

Variables	Category	*n*	%	$\chi^{2}$	*p*-Value
Total		1054	87.0		
Post-vaccination adverse reaction				22.192	<0.001
	No	931	88.8		
	Yes	123	73.2		
Age(years)				2.542	0.468
	<30	363	86.8		
	30~39	466	85.8		
	40~49	183	89.1		
	≥50	42	92.9		
Sex				0.241	0.624
	Male	165	88.5		
	Female	889	86.7		
Education				3.315	0.346
	Senior Secondary and below	71	87.3		
	Junior college	162	91.4		
	Undergraduate	709	86.2		
	Graduate	112	85.7		
Occupation				6.448	0.092
	Doctor	174	83.3		
	Nurse	670	86.6		
	Medical Technician	127	92.3		
	Others	83	89.2		
Professional titles				2.316	0.678
	Primary grade and below	465	86.7		
	Medium grade	359	86.1		
	Associate professor	86	88.4		
	Professor	47	93.6		
	Others	97	87.6		
Underlying disease				3.787	0.052
	Yes	121	81.0		
	No	933	87.8		

**Table 3:** Descriptive statistics and correlations among study variables (*n* = 1054).

Variables	Descriptive	1	2	3
1. Post-vaccination adverse reaction (Yes)	123 (11.7%)	1.00		
2. Decision regret	8.6 (±3.5)	0.14 ***	1.00	
3. Willingness to take the booster dose (Yes)	917 (87.0%)	−0.15 ***	−0.22 ***	1.00

Note: ***, *p* < 0.001. For the category variable, we used count (percentage) for the description, while for the continuous variable, we used mean (±sd).

**Table 4:** Testing of the mediating role of decision regret.

Variable	Model 1		Model 2		Model 3	
	OR	95%CI	B	95%CI	OR	95%CI
Independent variable						
Post-vaccination adverse reaction (No)						
Yes	0.37 ***	0.23~0.59	1.63 ***	0.98~2.28	0.46 **	0.29~0.74
Mediator						
Decision regret					0.84 ***	0.80~0.89
Controlled variable						
Age (<30)						
30~39	1.18	0.76~1.84	−0.30	−0.82~0.21	1.14	0.72~1.80
40~49	1.54	0.83~2.95	−0.58	−1.26~0.10	1.44	0.75~2.82
≥50	2.75	0.86~12.46	−0.20	−1.36~0.95	2.64	0.83~11.85
Sex (Male)						
Female	0.75	0.37~1.48	0.18	−0.55~0.91	0.78	0.38~1.55
Education (Senior Secondary and below)						
Junior college	3.10 *	1.01~9.56	−1.69 **	−2.83~−0.55	2.53	0.79~7.95
Undergraduate	1.95	0.70~5.17	−1.80 ***	−2.83~−0.78	1.55	0.54~4.23
Graduate	2.21	0.63~7.64	−2.21 **	−3.52~−0.89	1.56	0.43~5.62
Occupation (Doctor)						
Nurse	1.62	0.81~3.08	−0.54	−1.35~0.27	1.43	0.71~2.76
Medical Technician	3.21 *	1.34~8.32	−0.92 *	−1.81~−0.03	2.67 *	1.10~7.02
Others	2.33	0.82~7.21	0.28	−0.85~1.41	2.39	0.82~7.56
Underlying disease (No)						
Yes	0.56 *	0.33~0.97	−0.08	−0.75~0.59	0.54 *	0.32~0.94

Note: ***, *p*-value < 0.001; **, *p*-value < 0.01; *, *p*-value < 0.05. The outcome of Model 1 and 3 was willingness of taking the booster dose of COVID-19 vaccine (1 denotes “Yes”); the outcome of Model 2 was decision regret. Abbreviation: OR, odds ratio; CI, confidence interval; B, standardized beta regression coefficient.

## Data Availability

Data supporting the results of this study are available upon request from the authors.
